# Overcoming the Blood-Brain Barrier: Focused Ultrasound in Glioblastoma Treatment

**DOI:** 10.7759/cureus.82869

**Published:** 2025-04-23

**Authors:** Jonas Jurgaitis, Karina Mickeviciute, Valerija Jablonskiene

**Affiliations:** 1 Department of Neurosurgery and Neurology, Faculty of Medicine, Vilnius University, Vilnius, LTU; 2 Department of Neuroradiology, Faculty of Medicine, Vilnius University, Vilnius, LTU; 3 Department of Physiology, Biochemistry, Microbiology, and Laboratory Medicine, Faculty of Medicine, Vilnius University, Vilnius, LTU

**Keywords:** blood-brain barrier, focused ultrasound, glioblastoma, glioma, hifus, lifus

## Abstract

Despite recent advancements in neuro-oncology, glioblastoma remains one of the cancers with the poorest prognosis. Tumor recurrence and progression are driven by tumor stem cells and a high mutational burden. New potential treatment modalities are actively being explored. One promising approach is focused ultrasound (FUS), which involves converging ultrasound waves on a specific region of interest while avoiding damage to surrounding tissue. This mini-review highlights the current understanding of glioblastoma treatment resistance and positions FUS as a promising therapeutic option. We discuss existing clinical research utilizing FUS in four key treatment applications: (1) blood-brain barrier disruption, (2) histotripsy, (3) thermal ablation, and (4) sonodynamic therapy.

## Introduction and background

Glioblastoma (GBM) is the most prevalent and lethal form of primary brain cancer in adults, with an age-adjusted annual incidence ranging from 0.6 to 3.7 cases per 100,000 individuals [1]. It is most commonly diagnosed in older adults, with a median age at diagnosis of approximately 64 years. Incidence rates increase with age, reaching their highest levels between 75 and 84 years [2].

Despite treatment options like surgery, radiation therapy, tumor-treating fields, temozolomide (TMZ), and other chemotherapies such as lomustine and carmustine, long-term control of GBM remains difficult. Over the past 25 years, the Food and Drug Administration (FDA) has approved only two drugs specifically for high-grade gliomas (HGG): bevacizumab (BEV) and TMZ [3]. However, despite its widespread use, TMZ achieves only 20-30% of its plasma concentration in the central nervous system [4]. To maximize effectiveness, the current standard treatment for newly diagnosed GBM relies on a multimodal approach consisting of maximally safe surgical resection, followed by fractionated radiotherapy at a dose of 60 Gy over 30 sessions, combined with concurrent and adjuvant TMZ chemotherapy [5]. The extent of resection (EOR) remains a critical factor in determining overall survival (OS), with an EOR greater than 98% significantly associated with improved OS [6].

## Review

Materials and methods

This literature review was conducted to explore the relationship between blood-brain barrier (BBB) disruption and drug resistance in gliomas, with a focus on GBM. A comprehensive search was performed using databases including PubMed, Scopus, and Google Scholar. Keywords used in the search included "glioma", "glioblastoma", "blood-brain barrier disruption", "drug resistance", "efflux transporters", and "blood-brain tumor barrier". Studies that investigated both preclinical and clinical approaches to overcoming drug resistance were included. Relevant review articles, meta-analyses, and original research studies were carefully examined to extract data on mechanisms of BBB disruption, tumor adaptation, and strategies for improving drug delivery to gliomas. Inclusion criteria were peer-reviewed articles written in English that specifically addressed the role of the BBB in glioma development and drug resistance. Data from selected studies were analyzed to identify recurring themes and emerging strategies in the treatment of GBM.

BBB structure and its impact on drug delivery

**Figure 1:**
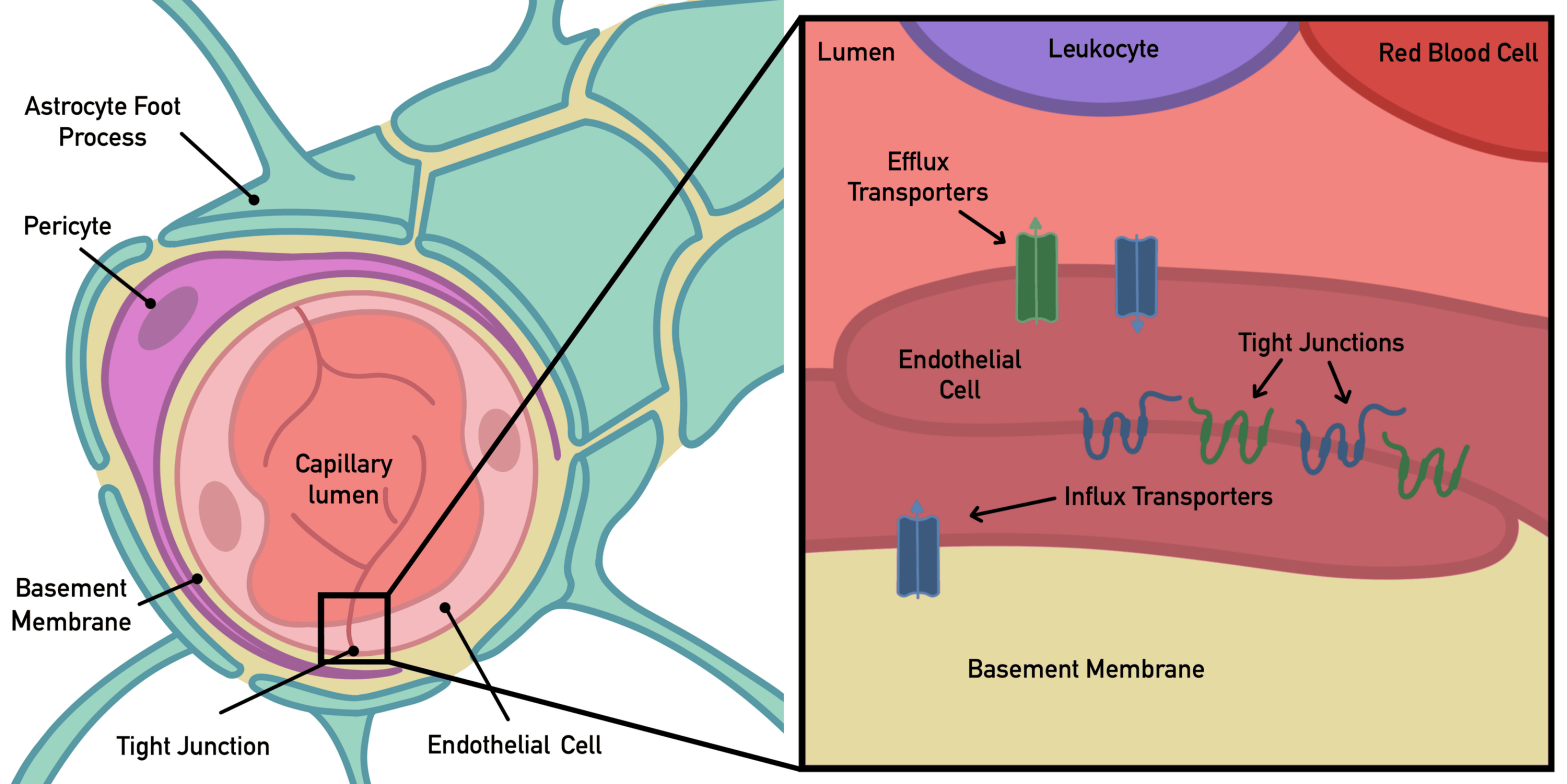
A schematic representation of the blood-brain barrier The illustration provides a schematic representation of the blood-brain barrier. The image on the left depicts a single neurovascular unit, while the image on the right offers a detailed view of the tight junctions and endothelial surface transporters. Adapted with permission from Martinez et al. [7].

The BBB is a limiting factor in effectively delivering drugs to the brain and has contributed to historically poor outcomes in brain cancer chemotherapy. Hence, it remains the primary obstacle to improving outcomes after drug administration. The BBB consists of specialized non-fenestrated endothelial cells, supported by astrocytic end-feet, surrounding pericytes, and a basal lamina (Figure 1).

These cells are connected by various junctions, including adherens junctions (such as vascular endothelial cadherin and platelet endothelial cell adhesion molecule-1), gap junctions (like connexin-37), scaffolding proteins (such as zona occludens-1), and tight junctions (including occludin and claudin-5) [8]. The BBB maintains brain integrity by regulating cerebral homeostasis and nutrient flow through various cell types using proteins and enzymes. It plays a crucial role in protecting the brain from harmful toxins, molecules, and inflammatory immune cells [9].

It has been observed that drugs larger than 400 Da often struggle to penetrate the BBB. Notably, lipophilic and uncharged molecules smaller than 400 Da, which are not bound to plasma proteins and lack membrane transporters, can diffuse freely. For instance, O2, CO2, water, and small lipid-soluble molecules easily cross the BBB through passive diffusion [10]. However, this threshold is not absolute, as peptides exceeding 600 Da can cross the BBB relatively easily and even larger molecules, such as the 7800 Da cytokine-induced neutrophil chemoattractant-1 (CNC1), can pass through via transmembrane diffusion [11,12]. The ability to cross the BBB is influenced by multiple factors, including charge, molecular weight, and hydrophobicity, leading to a complex, non-linear relationship between molecular size and BBB permeability [13]. Molecules can cross the BBB through various mechanisms, including active transport processes like carrier-mediated influx or efflux, as well as passive methods such as transmembrane diffusion and paracellular transport. Most molecules that successfully penetrate the BBB do so through either transmembrane diffusion or active transport [12].

BBB disruption in gliomas and their adaptation to drug resistance

During the early stages of glioma development, tumor cells attempt to mimic the characteristics of the BBB. As they grow and progress, they establish a new barrier known as the blood-brain tumor barrier (BBTB). The BBTB differs from the BBB due to an abnormal distribution of normal BBB cells, such as pericytes, a loss of astrocytic end-feet, neuronal dysfunction, varying permeability between the tumor core and its periphery, and elevated expression of proteins that promote drug efflux [14,15].

Drug resistance presents a major challenge in treating GBM, primarily due to the overexpression of drug resistance genes and the robust defense mechanisms of the BBB. The BBB is reinforced by efflux transport systems, such as P-glycoprotein, also known as multidrug resistance protein 1 or MDR1, which actively expels small molecules from endothelial cells into the bloodstream, impeding their delivery to brain tissue. Other efflux pumps, like breast cancer resistance protein (BCRP) and multidrug resistance-associated proteins (MRP), are critical defense mechanisms in the brain, functioning to protect against toxic xenobiotics [12]. Even when a potential therapeutic target is identified, variations in the expression of the target protein can diminish treatment efficacy. Therapy-resistant tumor cell populations often arise as sub-clonal cells with selectable traits that evolve in response to treatment. For instance, GBM develops resistance to TMZ by altering the expression of DNA alkylating proteins, DNA repair enzymes, and cell signaling pathways [16].

Studies have shown that HGG have areas with different levels of BBB disruption and vascular permeability [17,18]. Typically, the center of the tumor is more permeable compared to the area around the tumor and the surrounding brain tissue, which often retains an almost fully functional BBB [19]. Peripheral tumor areas located further from the center, with an intact BBB, protect invasive GBM stem cells. These cells, due to their high adaptability and metabolic flexibility, are major contributors to the recurrence and treatment resistance of GBM [20]. This challenges the common belief that the BBB is uniformly disrupted in gliomas. Evidence shows that only the central tumor regions benefit from increased drug accumulation due to a "leaky" BBB [21]. Neuwelt et al. introduced the concept of the "sink effect," which describes how chemotherapy agents that enter the central nervous system and reach the tumor tend to accumulate in areas of tumor necrosis. These necrotic regions act as "sinks," attracting and holding the administered drugs. As a result, the outer regions of the tumor, including the highly proliferative edges with cancerous cells, end up receiving lower concentrations of the drug [22-24].

A strategy to improve drug delivery to the brain, aiming for better treatment outcomes and potentially enhanced patient survival, includes administering high-dose intravenous chemotherapy, which may be combined with bone marrow transplantation, or using intra-arterial chemotherapy, paired with osmotic techniques to temporarily disrupt the BBB. Additionally, enhancing drug penetration into the brain can be achieved by modifying the drug itself or altering the properties of the BBB [25]. The future of treating brain tumors through the manipulation of the BBB is likely to depend not on a single technique but on the effective integration of multiple strategies. A promising approach could involve precisely opening the BBB with methods like osmotic disruption or focused ultrasound (FUS), followed by targeted intra-arterial drug delivery. This process, enhanced by real-time monitoring, might achieve therapeutic drug concentrations at the tumor site while minimizing harmful effects on the rest of the body [26].

Mechanisms and modalities of FUS

Because the BBB prevents achieving uniform, high drug concentrations needed for effective anti-tumor response, significant research has focused on methods to disrupt it. Since the impermeability of the BBB largely depends on tight junctions, most strategies aim to temporarily open these junctions. One such approach utilizes the ability to focus ultrasound waves on a specific region while sparing surrounding tissue, a technique known as FUS.

FUS is utilized in two forms based on intensity: high-intensity FUS (HIFUS) and low-intensity FUS (LIFUS). HIFUS is employed primarily for its lesional effects, where it can precisely target and ablate tissue, making it useful for destroying tumors or other unwanted tissue. On the other hand, LIFUS is used for its modulation effects, which involve influencing cellular processes or modulating brain activity without causing direct tissue damage [27].

Currently, FUS is primarily utilized in four key treatment applications, which are discussed below: (1) BBB disruption, (2) histotripsy, (3) thermal ablation, and (4) sonodynamic therapy.

BBB Disruption

One of the most promising uses of FUS is BBB disruption. Microbubbles play a critical role in converting acoustic energy into the mechanical effects necessary for opening the barrier. When subjected to acoustic pressure waves, these microbubbles expand and contract, generating fluid movement and shear stress in their immediate surroundings. This controlled back-and-forth movement, known as stable cavitation, occurs when ultrasound is delivered at intensities below the threshold for tissue damage, allowing the microbubbles to oscillate without collapsing. This oscillation within blood vessels can temporarily and reversibly disrupt the endothelial barrier, enabling the passage of both endogenous and exogenous molecules [28,29].

Moreover, when ultrasound activates microbubbles in direct contact with endothelial cells, it can induce a calcium wave that causes temporary cell contraction, leading to the opening of tight junctions [30]. Histological studies have further confirmed that tight junction protein expression increases following BBB opening, suggesting that these junctions are indeed disrupted, which enhances the permeability of the BBB. This process allows for a safer and more precise therapeutic effect, utilizing stable cavitation to interact with cells without causing tissue damage.

Carpentier et al. carried out a phase I clinical trial on patients with recurrent GBM who had the SonoCloud device implanted in the skull, specifically targeting both the tumor and the adjacent infiltrative regions, even those close to critical brain areas [31]. The trial involved 15 patients who underwent a total of 41 sonications at different acoustic pressure levels, in combination with carboplatin treatment. BBB disruption was successfully achieved in 28 of the 41 sonications, with higher acoustic pressures showing a stronger correlation. Safety evaluations demonstrated good patient tolerance, with no dose-limiting toxicities or acute adverse effects. Imaging conducted after sonication revealed no instances of acute hemorrhage, ischemia, or edema. Importantly, despite targeting critical brain regions, patients retained their ability to speak and move, with no detectable neuronal impact from the ultrasound.

In a study by Sonabend et al., 17 patients with recurrent GBM underwent treatment with albumin-bound paclitaxel following the implantation of the SonoCloud device and the disruption of the BBB [32]. The BBB disruption procedure led to temporary neurological deficits in several patients. Post-sonication, enhanced contrast MRI confirmed the successful opening of the BBB, indicated by varying degrees of gadolinium-based enhancement in the brain tissue. Notably, the BBB was restored within an hour after the procedure, around the same time that gadolinium was administered. Moreover, the levels of paclitaxel were significantly higher in the sonicated brain tissue compared to non-sonicated areas.

Beyond enhancing BBB permeability, FUS also exerts several secondary effects on brain tissue. These include activating microglia and triggering inflammatory responses, modifying cerebral blood flow, facilitating the clearance of amyloid-β and tau proteins, and reducing neuronal activity [33]. A study showed that administering high doses of FUS can stimulate the immune system within the tumor microenvironment by enhancing lymphocyte recruitment, potentially offering added advantages in cancer treatment [34]. Furthermore, there is evidence indicating that BBB disruption with FUS temporarily disrupts P-glycoprotein, which typically functions to expel various xenobiotic molecules into the bloodstream [35].

Histotripsy

Histotripsy is a non-invasive therapeutic technique that uses FUS to mechanically disrupt tissue at a microscopic level. It is a form of non-thermal HIFUS that relies on the mechanical effects of ultrasound rather than heat to achieve tissue destruction. When ultrasound waves are delivered at high acoustic intensities with short pulse durations, rather than in the continuous wave mode used in HIFUS-based tumor ablation, non-thermal effects become more prominent. Inertial cavitation generates shockwaves that liquefy cells, a process referred to as histotripsy [36,37].

Histotripsy offers two distinct methods: cavitation cloud histotripsy, which uses cavitation bubbles to precisely ablate tissue with minimal impact on surrounding areas, making it well-suited for sensitive regions like the brain and boiling histotripsy, which rapidly heats tissue to achieve faster ablation, particularly effective for treating larger or denser tumors, such as those in the liver [36].

FUS technology underlying histotripsy has been extensively investigated for various brain applications, including but not limited to BBB disruption. It has been used as an adjuvant to immunotherapy for brain malignancies and in treatments for conditions such as essential tremor, Parkinson's disease, Alzheimer's disease, epilepsy, and neuropathic pain. However, its potential as a therapeutic tool specifically for brain tumors or direct ablation of brain tissue has not been as thoroughly explored, with only a few studies, most of which remain at the preclinical stage [38].

Thermal Ablation

HIFUS-based tumor ablation has broadened its clinical use beyond functional neurosurgery, where it gained FDA approval in 2012 for procedures such as thalamotomy to treat essential tremor and is now being explored as an innovative treatment option for brain tumors [39]. High-frequency FUS waves target the intended tissue by causing rapid oscillation of fluid-filled microscopic cavities within the cells. The mechanical energy is converted into heat, raising the temperature at the focal point to 55-60°C, which results in thermal ablation by denaturing cellular proteins and organelles [40].

In 2010, McDannold et al. conducted a study that successfully demonstrated the ability to focus ultrasound beams transcranially into the brain in patients with GBM. They used FUS combined with MRI temperature imaging to monitor the thermal effects of ultrasound on brain tissue. This procedure was groundbreaking as it allowed them to visualize the heating of the targeted brain tissue in real-time. However, the study encountered significant challenges. Despite the ability to focus ultrasound beams, the FUS device they used had insufficient power to achieve the complete destruction of the tumor tissue. The trial was halted because the fourth patient experienced a fatal intracranial hemorrhage due to cavitation. Cavitation-induced damage in this case resulted in a fatal brain bleed [41].

Five studies explored the use of FUS for direct tumor ablation in treating HGG patients, with a total of 23 participants. Complication rates were relatively high, with more than one in four patients developing a hematoma at the FUS target site. This may be due to the use of early-generation devices and the limited knowledge available at the time regarding patient selection and risk factors. Despite this, only 6.4% of patients had to discontinue FUS treatment due to complications, indicating that the procedure was generally well-tolerated [42].

Moreover, hyperthermia may increase the sensitivity of glioma stem cells to radiation therapy through several mechanisms. It can inhibit DNA repair, making it harder for the cancer cells to fix the damage caused by radiation. Secondly, hyperthermia can activate both the innate and adaptive immune systems, promoting an immune response that targets the tumor more effectively. Also, it helps to reduce hypoxia, which typically promotes resistance to therapy, thus making the tumor cells more susceptible to radiation [43,44]. These combined effects may significantly enhance the effectiveness of radiation therapy in treating gliomas.

However, FUS-mediated hyperthermia currently has a limited role in the treatment of GBM due to several significant challenges. One of the main limitations is the need for a craniectomy. This is necessary because the HIFUS waves can be weakened by the skull and the bone itself can overheat, causing adverse effects. Without removing the skull bone, the ultrasound's energy is less effective, and heating could damage nearby healthy tissues [45]. Other challenges include the risk of ablation of healthy tissue along the path of the ultrasound waves due to insufficient precision. Despite these limitations in brain applications, HIFUS is clinically used successfully in the treatment of tumors in other parts of the body, such as the pancreas, breast, prostate, and uterine fibroids [46]. The focus remains on improving HIFUS technology for more precise, safer applications in the brain, while it continues to see success in treating other types of tumors and conditions.

Sonodynamic Therapy

Sonodynamic therapy is an emerging treatment approach for glioma that functions similarly to photodynamic therapy but with key advantages. The two key components of sonodynamic therapy, namely, sonosensitizer and FUS, are individually nontoxic. However, when used together, they combine to induce cellular damage and death, effectively targeting and destroying cancer cells while minimizing harm to surrounding healthy tissue [26,47].

In photodynamic therapy, a photosensitizer is activated by light to produce reactive oxygen species, which then induces cytotoxic effects on cancer cells. However, photodynamic therapy's effectiveness is limited to superficial tumors due to the poor penetration of light into deeper brain tissues. Sonodynamic therapy addresses this limitation by using low-intensity ultrasound, which can penetrate more than 8 cm beyond the focus point, allowing it to reach deeper tissues. In sonodynamic therapy, ultrasound is combined with sonosensitizers that make cancer cells more susceptible to sound-induced damage, thereby enhancing the therapy's precision and reducing potential side effects. Sonodynamic therapy does not require invasive procedures such as craniotomy or the stereotactic placement of light fibers, making it a less invasive and more versatile option [48].

A variety of compounds have been researched for use in sonodynamic therapy, including porphyrins, chlorins, and phthalocyanines. Among these, the most effective have been hydrophobic compounds that can integrate into the cellular membrane, enhancing their ability to generate therapeutic effects [49].

5-Aminolevulinic acid (5-ALA) is an FDA-approved agent used during surgery to enhance the visualization of gliomas through fluorescence-guided techniques. Recently, 5-ALA has gained attention for its potential in clinical applications beyond imaging, and it is currently being explored as a sonosensitizing agent in sonodynamic therapy. When utilized in sonodynamic therapy protocols, tumor cells metabolize 5-ALA into protoporphyrin IX (PpIX), which subsequently produces reactive oxygen species and induces cell death, offering a promising approach to cancer treatment [50].

Multiple studies have demonstrated the synergistic effects of combining FUS with 5-ALA. For instance, in 2020, Sheehan et al. provided evidence of enhanced cell death using GBM as the tumor model. Their research showed that sonodynamic therapy combining HIFUS and 5-ALA led to a 47% reduction in cellular viability and a 238% increase in caspase-3 activity in both C6 and U87 GBM cell lines [51]. Additionally, a study by Ohmura et al. compared survival outcomes in mice with gliomas treated with 5-ALA and FUS, FUS alone, or 5-ALA alone. The results indicated that the combination of 5-ALA and FUS significantly increased reactive oxygen species levels in the treated mice compared to untreated controls, suggesting a more effective therapeutic outcome [50].

## Conclusions

Despite advancements in glioma treatment, the BBB remains a critical obstacle to significantly improving survival rates for these patients. FUS techniques offer promising solutions through various approaches. While each modality shows potential, particularly in enhancing treatment specificity and minimizing damage, challenges such as drug resistance, safety concerns, and variable patient outcomes persist. Further clinical trials and research are essential to optimize these techniques and expand their use in the treatment of brain tumors and other neurological conditions.
